# Microbiology of Prosthetic Joint Infections: A Retrospective Study of an Italian Orthopaedic Hospital

**DOI:** 10.3390/antibiotics13050399

**Published:** 2024-04-26

**Authors:** Virginia Suardi, Daniele Baroni, Abdelrahman Hosni Abdelhamid Shahein, Valentina Morena, Nicola Logoluso, Laura Mangiavini, Antonio Virgilio Pellegrini

**Affiliations:** 1 IRCCS Ospedale Galeazzi–Sant’Ambrogio, 20157 Milan, Italy; virginia.suardi@grupposandonato.it (V.S.); nicola.logoluso@grupposandonato.it (N.L.); antonio.pellegrini@grupposandonato.it (A.V.P.); 2 Department of Orthopedics and Traumatology, Alessandro Manzoni Hospital, 23900 Lecco, Italy; da.baroni@asst-lecco.it; 3 Residency Program in Orthopaedic and Traumatology, University of Milan, 20122 Milan, Italy; abdelrahman.shahein@unimi.it; 4 Infectious Disease Unit, Alessandro Manzoni Hospital, 23900 Lecco, Italy; v.morena@asst-lecco.it; 5 Department of Biomedical Sciences for Health, University of Milan, 20133 Milan, Italy

**Keywords:** PJI, bacteria, microbiology, antibiotic prophylaxis

## Abstract

The most frequent cause of periprosthetic infections (PJIs) is intraoperative contamination; hence, antibiotic prophylaxis plays a crucial role in prevention. Modifications to standard prophylaxis can be considered if there is a high incidence of microorganisms resistant to current protocols. To date, very few studies regarding microbial etiology have been published in Italy. In this single-center, retrospective study conducted at IRCCS Ospedale Galeazzi-Sant’Ambrogio in Milan, we analyzed hip, knee, and shoulder PJIs in patients undergoing first implantation between 1 January 17 and 31 December 2021. The primary aim was to derive a local microbiological case history. The secondary aim was to evaluate the adequacy of preoperative antibiotic prophylaxis in relation to the identified bacteria. A total of 57 PJIs and 65 pathogens were identified: 16 S. aureus, 15 *S. epidermidis*, and 10 other coagulase-negative staphylococci (CoNS), which accounted for 63% of the isolations. A total of 86.7% of *S. epidermidis* were methicillin-resistant (MRSE). In line with other case reports, we found a predominance of staphylococcal infections, with a lower percentage of MRSA than the Italian average, while we found a high percentage of MRSE. We estimated that 44.6% of the bacteria isolated were resistant to cefazolin, our standard prophylaxis. These PJIs could be prevented by using glycopeptide alone or in combination with cefazolin, but the literature reports conflicting results regarding the adequacy of such prophylaxis. In conclusion, our study showed that in our local hospital, our standard antibiotic prophylaxis is ineffective for almost half of the cases, highlighting the importance of defining specific antibiotic guidelines based on the local bacterial prevalence of each institution.

## 1. Introduction

Periprosthetic joint infections (PJIs) represent a severe surgical complication that causes significant morbidity and mortality in patients, as well as high social costs. 

Prophylactic administration and scheduling of antibiotics preoperatively and the use of laminar air flow during surgery are some of the measures used to lower the incidence of PJIs [1,2,3]. In addition to the above, many other factors are being investigated to reduce the risk of infection: patient preparation (pre-operative antiseptic wash, trichotomy, optimization of glycaemia in diabetic patients), limiting traffic in the operating theatre, and the use of adhesive drapes or pulsed washing devices. Nevertheless, the incidence of PJI after primary total hip arthroplasty (THA) ranges from 0.9% to 2.0%, and all-cause mortality related to PJI has been reported to be 5% after 1 year and 20% after 5 years [4]. Significant healthcare costs are connected with PJI, as are the short-term burdens of prolonged sick leave, repeated surgery, and pain [5].

Since there is currently no single test that can diagnose PJI with 100% accuracy, a combination of imaging, microbiological and histological tests, serum and synovial fluid indicators, and clinical evaluation is needed to make the diagnosis. Furthermore, even the most modern and sophisticated scores may come back as “inconclusive” due to the vast range of clinical presentations that periprosthetic joint infections may show, from acute, high-grade to subclinical, low-grade.

Although, to date, many diagnostic criteria have been proposed, the ones from the 2018 International Consensus Meeting of Philadelphia [6] are widely used, as well as at our institution.

The choice of treatment must be personalized for each individual patient and depends on different factors. In addition to the virulence and resistance profile of pathogens (when known) and the timing of the onset of PJI, patient-related factors must also be considered, including bone stock, soft tissue quality, eligibility for one or more surgical procedures [7], and eligibility for prolonged antibiotic therapies. If surgery is not an option, long-term suppressive antibiotic therapy is a possibility.

The current surgical treatment consists of one or two phases of revision arthroplasty, which is typically reserved for chronic stages, while “debridement, antibiotics, and implant retention" (DAIR) is traditionally performed early after the development of symptoms. Furthermore, in extreme circumstances, arthrodesis, amputation (knee), resection arthroplasty (hip), and definitive articulating antibiotic spacer (shoulder) may be carried out [8].

This difference is explained by biofilm formation in the case of the foreign body [9,10]: complex populations of microorganisms embedded in an extracellular matrix that develop on surfaces. Microorganisms can live in free form or in a consortium of different or the same species, called biofilm. Biofilm is an ordered and arranged group of microorganisms living within an extracellular polymeric substance matrix, produced by them, and showing variation in terms of growth rate and gene expression when compared to their planktonic form [11]. Biofilms have a heterogeneous structure composed mainly of microbial cells (10–25%) and extracellular matrix (75–90%), which contains water, polysaccharides, proteins, DNA, and RNA [12]. Most of the polysaccharides are heterogeneous, while some are homogeneous, as are cellulose, sucrose-derived fructans, and glucans [13]. The barrier function of this biofilm environment reduces the effectiveness of antimicrobials and host defenses. In addition, pathogens buried deep within the biofilm have a typically slow metabolic rate that makes accurate culture identification more complex, requiring procedures such as sonication, longer culture times, and enrichment of breeding grounds. Different microorganisms may show different interactions with the extracellular matrix. It has been demonstrated that surfactants and modulins play a role in staphylococcal biofilm maturation [14]. Mature biofilm may rupture actively (motility and extracellular matrix degradation-dependent dispersion). The main factors responsible for the dispersion of mature biofilm include an outgrown population, intense competition, a lack of nutrients [15], enzyme action that causes alginate digestion in *Pseudomonas* spp. [16], variations in environmental conditions, as well as up/downregulation of genes promoting motility [17]. *Pseudomonas aeruginosa, S. aureus,* and *S. epidermis* together account for over 75% of the biofilms present in medical equipment. *S. epidermis* and *S. aureus* are the most prevalent bacteria that create biofilms.

Due to the complexity of these diseases, the creation of a multidisciplinary team for the management of PJIs, including orthopaedic surgeons, infectious disease specialists, clinical microbiologists, and pharmacologists, can represent an added value to improving the clinical outcome in this scenario [18]. In addition, radiologists, nuclear medicine specialists, and other surgeons, especially the plastic surgeon, can also play a key role in selected cases.

Microorganisms introduced during surgery account for the majority of PJIs that arise within a year after surgery. The prosthesis or periprosthetic tissue may become contaminated either directly or by aerosolized means. Microorganisms populate the implant’s surface once they come into contact with it. The minimal inoculum of microorganisms required to cause infection in the presence of the prosthetic material is a crucial component in this process [5]. In a rabbit model after hip hemiarthroplasty, for instance, < 102 CFU of *S. aureus* are required to develop infection, as opposed to 104 CFU in the absence of an implant [19].

These infections may also present after several years from the primary surgery due to haematogenous seeding, but the main cause is usually direct contamination during the implantation. Hence, prevention is mandatory in order to reduce the risk of infection during surgery. In 2021, the Italian Society of Orthopaedics and Traumatology (SIOT) released its latest guidelines regarding PJI prevention. As it concerns antimicrobial preoperative prophylaxis, SIOT reports that, because of the relatively low incidence of PJI, comparative studies hardly reach a sufficient number of patients to surely demonstrate the superiority of a defined antimicrobial drug. The antimicrobial choice is mainly based on the presumption of colonization and on the pathogens commonly reported in that type of surgery [20]. First- and second-generation cephalosporins have their own bactericidal activity on gram+ and some gram- bacilli, as well as good penetration into bone, synovial tissues, and muscular tissues with low systemic toxicity. Cefazolin, the most studied and tested in clinical studies [20], represents the recommended prophylaxis in other guidelines [21].

According to SIOT guidelines, different standard prophylaxis could be considered in cases of documented high local prevalence of bacteria resistant to standard protocols. Similarly, according to American Academy of Orthopaedic Surgeons (AAOS) guidelines [21], antibiotic selection should reflect the antibiogram of the individual institution, the individual risk factors of the patient, and the multidisciplinary support of institutional infection control experts.

Resistance patterns are different among regions and geographical areas. For instance, according to the 2017 European Centre of Disease Control (ECDC) report, in Italy, there is a high incidence of methicillin-resistant bacteria [22], and in general, the presence of methicillin resistance among isolated *S. aureus* in many Mediterranean countries is far greater than in those isolated in Scandinavian countries. So, the microbiological reports of periprosthetic infections in other European countries are not automatically applicable to the Italian territory due to differences in their microbiological environments. Reliable national and local data are therefore needed to optimize protocols for preoperative prophylaxis. To date, with regard to Italian microbiological reports, very few studies have been published [23].

Knowing the local microbiology of the hospital and/or of the geographical area can be useful in guiding the choice of empiric antibiotic therapy as well as in the choice of preoperative antibiotic prophylaxis.

The main purpose of our study was to produce a local microbiological report, analyzing cases of early or delayed periprosthetic joint infection of total hip, knee, and shoulder arthroplasty procedures performed at IRCCS Ospedale Galeazzi–Sant’Ambrogio. The secondary purpose was to evaluate the adequacy of preoperative antibiotic prophylaxis in relation to the antibiotic susceptibility profiles of the identified microorganisms.

## 2. Results

A total of 182 patients were found in both the surgical records: a list of 17,256 primary hip, knee, and shoulder arthroplasties and another list of 1294 surgical procedures for prothesis-related complications.

After applying the selection criteria and collecting data, we identified 57 cases of PJI in 57 patients. The median age of patients was 70 years (interquartile range of 61–78 years). Infections involved the hip in 26 cases, the knee in 23 cases, and the shoulder in 8 cases.

The onset of the infection was early in 27 cases and delayed in 30.

A total of 65 pathogens were identified, with 33 in early-onset cases and 32 in delayed-onset cases, and with 58 in hip and knee cases (grouped together) and 7 in shoulder cases. In 10 cases (17.5%) of confirmed periprosthetic infections, cultures were negative.

Polymicrobial flora was identified in 14 (24.5%) cases, of which 9 had early onset and 5 had delayed onset. Hence, in our case studies, polymicrobial infections represent 33.3% of early infections and 16.7% of delayed infections. In detail, as follows:

Early polymicrobial:

- MSSA (Methicillin-susceptible *Staphylococcus aureus*) + *Enterococcus faecalis*
- MSSA + *Enterococcus faecalis*
- MRSE (Methicillin-resistant *Staphylococcus epidermidis*) + *Finegoldia magna*
- MRSE + *Finegoldia magna*
- MRSE + MSSE (Methicillin-susceptible *Staphylococcus epidermidis*)
- MRSE + *S. warneri*
- MRSE + *S. lugdunensis*
- *Streptococcus mitis* + *Cutibacterium acnes*
- *Corynebacterium amycolatum* + *Corynebacterium jeikeium* + *Morganella morganii*

Delayed polymicrobial:

- MRSE + *Cutibacterium acnes*
- MRSA (Methicillin-resistant *Staphylococcus aureus*) + *Staphylococcus saprophyticus*
- MSSA + MRSE
- MRSE + *Staphylococcus lugdunensis*
- MSSA + *Staphylococcus capitis*

Table 1 shows the bacteria that were found, divided by onset and involved joint. Globally, in the 57 patients included in the study, we identified 16 *S. aureus*, 15 *S. epidermidis*, and 10 other Coagulase-negative *Staphylococci* (CoNS), which together accounted for 63% of the isolated bacteria. Among the remaining gram+ bacteria, we identified 4 *Streptococcus* spp., 3 *Corynebacterium* spp., 3 *Enterococcus faecalis*, 5 *Cutibacterium acnes*, and 2 *Finegoldia magna*. Among the 7 gram- microorganisms (10.8%), there are 2 *Enterobacter cloacae,* 2 *Proteus mirabilis*, 1 *Escherichia coli*, 1 *Pseudomonas aeruginosa*, and 1 *Morganella morganii*.

**Figure 1:**
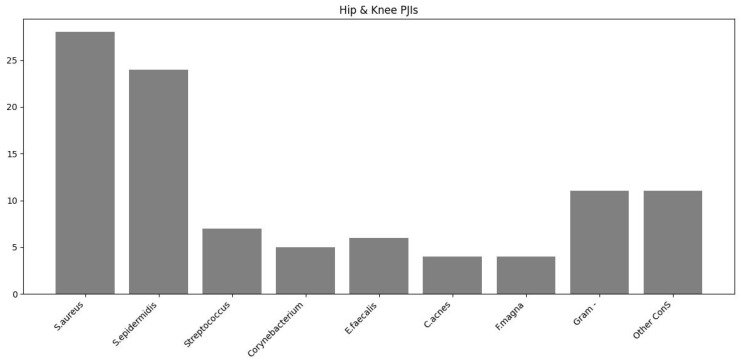
The prevalence rates of bacteria isolated in cases of periprosthetic hip and knee infections.

**Figure 2:**
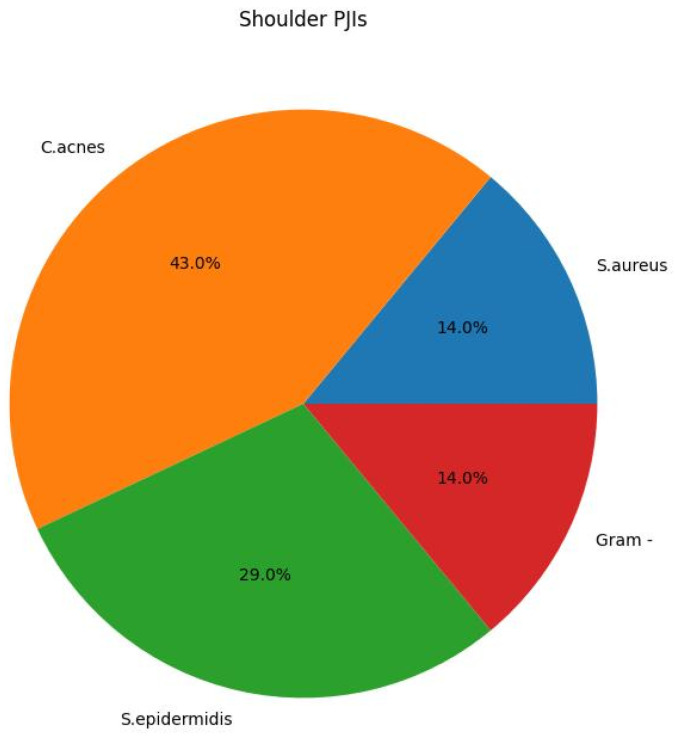
The prevalence rates of bacteria isolated in cases of periprosthetic shoulder infection.

**Figure 3:**
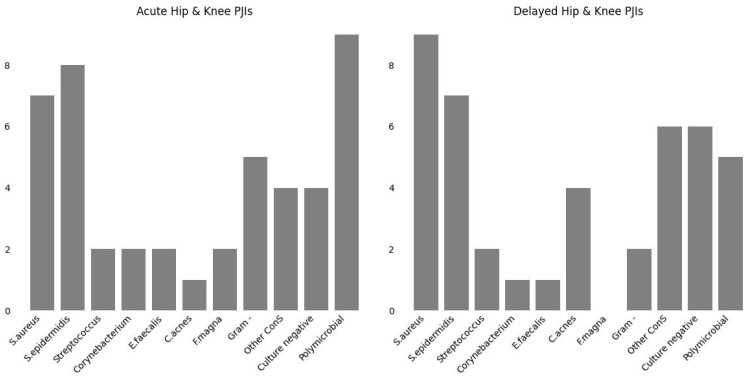
Cumulative number of bacteria in early vs. delayed PJI.

In the following diagrams (Figure 1, Figure 2 and Figure 3), the prevalence rates of bacteria isolated in cases of periprosthetic hip and knee vs. shoulder infection and the cumulative number of bacteria in early vs. delayed PJI are reported.

In detail (Table 2), for *Staphylococcus* spp., we show the number of bacteria resistant to tested antimicrobial drugs, as well as cefazolin (resistance inferred from oxacillin).

In our series, the prevalence of methicillin resistance was 18.8% for *S. aureus*, while a high frequency was found among *S. epidermidis* (86.7%). These MRSEs also showed high frequencies of resistance to levofloxacin (9/15), tetracycline (9/15), gentamicin (7/15), cotrimoxazole (5/15), and clindamycin (8/15).

## 3. Discussion

Periprosthetic joint infections represent a serious complication with significant morbidity and mortality. The correct identification of the pathogen and the appropriateness of antibiotic therapy are two key elements in the treatment of PJI. Knowing the local microbiology of the hospital and/or the geographical area can be useful in guiding the choice of empiric antibiotic therapy as well as in the choice of preoperative antibiotic prophylaxis. The main purpose of our study was to produce a local microbiological report, analyzing cases of early or delayed periprosthetic joint infection regarding total hip, knee, and shoulder arthroplasty procedures performed at IRCCS. The secondary purpose was to evaluate the adequacy of preoperative antibiotic prophylaxis in relation to the antibiotic susceptibility profiles of the identified microorganisms. In the literature, there is a substantial paucity of microbiological data concerning Italian case series. In a study [25] conducted at IRCCS, published in 2017, Drago et al. reported a series of 429 patients with late hip or knee PJI, of whom only 30% had undergone their first arthroplasty procedure at IRCCS.

The authors reported a prevalence of staphylococci in 66.6% of cases. Among the 341 isolated staphylococci cases, they stated a prevalence of methicillin resistance in the hip and knee of 23.3–21.7% for *S. aureus*, 68.9–64.4% for *S. epidermidis,* and 41.8–29.2% among non-epidermidis CoNS. In our series, we found a percentage of MRSA of 18.8% and a percentage of MRSE of 87.6%; among CoNS, however, methicillin resistance was present globally in 30%, or more specifically in 2/4 *S. lugdunensis* and 1/1 *S. warneri*. In another Italian study published in 2011, concerning infections associated with orthopaedic implants [26], Montanaro et al. conducted a large study on a collection of 1027 isolates obtained in the period between 2000 and 2003 from 699 orthopaedic patients bearing infections and reported a staphylococci frequency of 78.1% (*S. aureus* 31.7%, *S. epidermidis* 39.0%). Carrega et al. [27] reported in 2008 the microbiological diagnoses of 141 patients with early, delayed, or late periprosthetic infection. During the 35-month study period, 228 patients with prosthetic joint infections were retrospectively evaluated. The etiology of the infection was established by means of cultures performed during surgical revision of the infected prosthesis, sterile needle aspirates, or swabs (3 samples) taken deeply in the fistulous tract. Isolated bacteria were 73% staphylococci, 11% gram+, and 16% gram−. They also reported 16% polymicrobial infections.

Mussa et al. [23] performed a retrospective study at a single center with records of patients treated for primary PJIs of the knee or hip from 2011 to 2018. Infections were diagnosed according to IDSA and MSIS criteria, and all patients underwent blood cultures, synovial fluid cultures, periarticular biopsies, and prosthesis sonication. The authors analyzed microbiological data from 51 PJIs with different onsets and related to different joints. In their series, *S. aureus* accounted for 27.5%, other CoNS for 13.7%, and, in 33% of cases, negative cultures were reported. In addition, the MRSA rate was 28%. In 2017, in the annual survey on antibiotic resistance [28], the ECDC reported an MRSA rate of 33.9% among *S. aureus* isolated in Italy, compared to a European average of 16.9%. The percentage of MRSA in our series (18.8%) is very similar to the European one, rather than the Italian percentage. Regarding periprosthetic shoulder infections, our series (7 cases) does not allow us to draw conclusions, but the percentage of *C. acnes* was 43%, a value not far from the 38.9% reported by Nelson et al. [29] in a systematic review including 324 patients from 16 studies. In our study, 63% of the bacterial isolates are different types of staphylococci, similarly to the study by Drago et al. and apparently inferior to the results of other Italian studies. The most interesting finding is a very high percentage of MRSE, 86.7%, among the *S. epidermidis*. In addition, these *S. epidermidis* showed high rates of resistance to levofloxacin, tetracycline, gentamicin, cotrimoxazole, and clindamycin, many of which represent antibiotics commonly used in the treatment of PJI. This association of resistance between different antibiotics has already been described in the literature [30,31,32,33]. Mussa et al. reported [23] a high frequency of resistance to fluoroquinolones, tetracyclines, and cotrimoxazole among CoNS. Fluoroquinolones, tetracyclines, and cotrimoxazole are oral antibiotics known to have good penetration into bone tissue [34]. Gentamycin is commonly contained in or added to cements used both to stabilize implants and as a material for antibiotic-loaded spacers in two-stage procedures. Clindamycin, instead, is frequently used for preoperative prophylaxis as an alternative to cefazolin in patients with a reported allergy to beta-lactams. Rifampicin, on the other hand, well known for its anti-biofilm properties [35], was potentially ineffective only in 1 isolate of *S. aureus* and 3 of *S. epidermidis*. In light of our results, the only antibiotics to which no isolated bacteria were resistant were vancomycin, teicoplanin, and linezolid. On the other hand, daptomycin, also a known therapeutic option for periprosthetic infections, was ineffective in only two strains of *S. epidermidis*. In staphylococci, including coagulase-negative, the expression of an additional penicillin-binding protein (PBP2a) leads to complete resistance to beta-lactams, i.e., penicillins, cephalosporins, and carbapenems, with the only exception represented by the most recent cephalosporins with anti-MRSA activity, namely ceftaroline and ceftobiprole. The expression of this PBP2a is associated with the presence of the mecA gene [31], found in 85% of the *S. epidermidis* of that series. At our institution, the standard preoperative prophylaxis is cefazolin 2 g ev 30 minutes before the incision. Alternatives, in cases of a reported allergy to beta-lactams, are clindamycin 600–900 mg and vancomycin 1 g. Since resistance to oxacillin (used to define methicillin-resistant staphylococcal strains) also confers resistance to cefazolin, this antibiotic may be ineffective in preventing infections acquired in the operating room in case of contamination with methicillin-resistant bacteria [36]. In addition, resistance to cefazolin was also assumed for *Streptococci*, from resistance to penicillin [37], with therefore only one resistant Streptococcus (*S. mitis*).

In addition, all *E. faecalis*, *E. cloacae*, *P. aeruginosa,* and *M. morganii* are intrinsically resistant to cefazolin. Also, *E. coli*, which may be potentially sensitive to cefazolin, was resistant to this antibiotic as a producer of extended-spectrum beta-lactamase (ESBL+). Ultimately, we can conclude that at least 29 of the 65 isolated bacteria, or 44.6%, were resistant to our standard antibiotic prophylaxis. These infections could potentially be prevented by using vancomycin or teicoplanin as preoperative prophylaxis [36], but with the disadvantage that these antibiotics, when used alone, do not prevent the infection caused by some gram− bacteria, such as *E. coli*, *P. mirabilis,* and other Enterobacteriaceae. Another alternative would consist of a double antibiotic prophylaxis with cefazolin and vancomycin or teicoplanin, but the literature reports conflicting results [38,39]. In addition, double prophylaxis with double antibiotics (vancomycin and beta-lactam) has been associated with a possible occurrence of acute renal failure and *C. difficile* infections compared to prophylaxis with a single antibiotic in cardiac surgery [40], but not in other specialties, orthopaedics included. Among the gram- bacteria present in our series, the only antibiotics that have never been associated with resistance were amikacin and meropenem. However, given the small number of cases, we cannot draw any definitive conclusion on their efficacy in prophylaxis.

Our study has potential biases. First of all, in view of the method used to derive the case studies, it is not possible to calculate the incidence of periprosthetic infections. In consideration of the inclusion criteria, we may have wrongly included patients or microorganisms because of false positive cultures, but it is likely that the number of cases and pathogens included in the study is underestimated due to the following possible biases:

- false negative cultures
- diagnostic delays
- treatment delays
- patients undergoing surgery close to the time limits of the study (PJIs occurring on prostheses implanted before 2017 and possible PJIs manifested in 2022)
- incorrect/different ICD-9 encoding in surgery records
- PJIs treated in other hospitals
- possible deaths due to PJI

## 4. Materials and Methods

### 4.1. Setting

This paper presents a single-center, retrospective observational study concerning cases of periprosthetic infection of the hip, knee, and shoulder, early and delayed onset, in patients who underwent first prosthetic implantation at the IRCCS Ospedale Galeazzi-Sant’Ambrogio (Italy). Our institute is an orthopaedic hospital where over 3500 arthroplasty procedures are performed by different orthopaedic teams every year.

At our institution, the standard preoperative prophylaxis is cefazolin 2 g ev 30 min before the incision. Alternatives, in cases of a reported allergy to beta-lactams, are clindamycin 600–900 mg and vancomycin 1 g.

### 4.2. Selection Criteria

Inclusion criteria were:

1. Patients who underwent both

- hip, knee, or shoulder arthroplasty
- a second surgical procedure for diagnosed or suspected PJI or other prosthetic joint-related complications where intraoperative cultures were collected

2. Both procedures were performed at the Galeazzi Orthopaedic Institute
3. Both procedures were performed between 01/01/17 and 31/12/21

To identify the cases that could potentially be included, we obtained two lists of surgical records from the software for the compilation and archiving of surgical acts in use at IRCCS Ospedale Galeazzi-Sant’Ambrogio(HDocs–SB Italia, Garbagnate Milanese, Italy), regarding:

(1) hip, knee, or shoulder arthroplasty (ICD-9 81.51, 81.54, 81.80)
(2) Any surgery related to the diagnosis of infection or other complications related to joint replacements (ICD-9 966.66, 966.67, 966.77)

Then, we cross-checked the lists to identify the patients present in both lists, and we examined the medical records of the identified patients to apply the following exclusion criteria:

- Revision surgeries not related to PJI (e.g., aseptic loosening confirmed by negative intraoperative cultures)
- PJI occurring more than 1 year after the index surgery
- Patients undergoing the two surgical procedures at different joints
- For the purposes of our study, we have classified PJI by onset, as follows:
- Early: less than 4 weeks from surgery
- Delayed: between 4 weeks and 1 year
- Late: more than 1 year (excluded from the study)

We decided to divide between early and delayed PJI following the Tsukayama classification [41], as timing is crucial to performing the DAIR (Debridment, Antibiotics, and Implant Retention) procedure. Moreover, with regard to the threshold between delayed and late infections, we have chosen to set it at 1 year instead of 2, with the aim of increasing the chances that the isolated pathogens were actually acquired during the intervention or during the hospital stay.

### 4.3. Microbiological Testing

For each patient included, we considered pathogens isolated from intraoperative samples and preoperative arthrocentesis.

Intraoperative samples included osteoarticular fragments, synovial membranes, and soft tissue biopsies collected during surgery. In addition, preoperative arthrocentesis cultures were also considered. Wound swabs were not included since they are not reliable samples due to the high contamination rate from the skin microflora.

Microbiological testing was performed according to standard procedures at our institution. Before culture, samples were treated with 0.1% *w*:*v* Dithiothreitol to free pathogens from the biofilm.

Implants and periprosthetic tissues were treated with a 0.1% Dithiothreitol solution, as previously described [42,43]. Briefly, DTT solution was added to each sample to fully cover the entire surface of the sample. After agitation for 15 min at room temperature, DTT eluate was collected in sterile tubes and centrifuged at 2800× *g* 10 min. Then supernatant in excess was discharged, and the pellet was resuspended in a small volume of supernatant. Aliquots of bacterial suspension were finally seeded onto Chocolate agar, MacConkey agar, Mannitol salt agar, and Sabouraud agar and inoculated into Brain Heart Infusion broth and Thyoglycollate broth. Plates and broths were incubated for 48 h and 15 days at 37 °C in proper conditions, respectively. Broths were daily checked for microbial growth, and in the case of turbidity, an aliquot was plated on Blood agar and Schaedler agar to support the growth of aerobes and anaerobes, respectively. Aliquots from the Brain Heart infusion broth showing turbidity were plated on Blood agar, and those from the Thioglycolate broth were plated on Schaedler agar.

Microbial identification and antimicrobial susceptibility testing were carried out on a Vitek2 system.

### 4.4. Pathogens and Resistance Profiles

When applicable, for each identified bacterium species, we inferred susceptibility to cefazolin using the “EUCAST 2022 Breakpoint tables” [37] and the “Sanford guide to antimicrobial therapy” (51st edition, year 2021) [44] with the support of an infectious disease specialist. In detail, resistance to cefazolin was inferred from:

- resistance to oxacilline in *Staphilococcus* spp.
- resistance to penicillin in *Streptoccus* spp.
- resistance to cefotaxime in *E. coli* and *P. mirabilis*

*E. faecalis*, *E. cloacae*, *P. aeruginosa,* and *M. morganii* are intrinsically resistant to cefazolin.

## 5. Conclusions

Our study reports an Italian single-center microbiological series regarding early and delayed PJI, therefore probably acquired during the surgical procedure or hospital stay. Despite the possible biases, we found:

- a predominance of staphylococcal infections and, in general, a predominance of gram+ over gram−
- a percentage of MRSA lower than the Italian average and similar to the European one
- a high percentage of MRSEs, which exhibit high rates of resistance to other classes of antibiotics, as already highlighted in the literature

It is very important to know the local microbiology and resistance patterns. We believe that our case series provides a good representation of the bacterial flora present in our institute, and we have estimated that at least 44.6% of the isolated bacteria were resistant to our standard preoperative antibiotic prophylaxis. Nevertheless, evidence on the potential efficacy of glycopeptides in preoperative prophylaxis is still lacking, so for now we can reserve their use in patients with a history of colonization/infection by MRSA or related risk factors. In conclusion, although SIOT guidelines state that it is possible to change the standard prophylaxis in consideration of particular local microbiological conditions, a threshold for which this change would be effective in reducing the rate of infections remains to be defined. It is our opinion that further studies with a prospective randomized control design with the aim of comparing standard cefazolin with other preoperative prophylaxis, given an already known local microbiological situation, could show the superiority of other antibiotics compared to cefazolin as the prevalence of cefazolin-resistant bacteria increases. In this scenario, it would be possible to define specific antibiotic preoperative prophylaxis protocols based on the local bacterial prevalence of each institution.

## Figures and Tables

**Table 1:** Summary table of the bacteria isolated in our case series, divided by onset and location.

		Total	Early	Delayed	Hip and Knee	Shoulder
Gram+	*S. aureus*	16	7	9	15	1
	*S. epidermidis*	15	8	7	13	2
	*S. lugdunensis*	6	3	3	6	
	*S. saprophyticus*	1		1	1	
	*S. capitis*	1		1	1	
	*S. caprae*	1		1	1	
	*S. warneri*	1	1		1	
	*S. pneumoniae*	1	1		1	
	*S. mitis*	1	1		1	
	*S. agalactiae*	1		1	1	
	*S. equisimilis*	1		1	1	
	*C. amycolatum*	1	1		1	
	*C. jeikeium*	1	1		1	
	*C. striatum*	1		1	1	
	*E. faecalis*	3	2	1	3	
	*C. acnes*	5	1	4	2	3
	*F. magna*	2	2		2	
Gram−	*E. cloacae*	2	1	1	1	1
	*P. mirabilis*	2	2		2	
	*E. coli*	1		1	1	
	*P. aeruginosa*	1	1		1	
	*M. morganii*	1	1		1	
	Total	65	33	32	58	7

**Table 2:** Staphylococcal resistance profile. CoNS (Coagulase-negative *Staphylococcus*). We included in the table anti-Staphilococcical antibiotics commonly used in the treatment of bone and joint infections, which are routinely tested by our microbiology laboratory. All of these have good bone and synovial fluid distribution [24].

Antibiotic	*S. aureus*	*S. epidermidis*	Others CoNS
Oxacillin	3/16	13/15	3/10
Levofloxacin	2/16	9/15	1/10
Daptomycin	0/16	2/15	0/10
Rifampicin	1/16	3/15	0/10
Vancomycin	0/16	0/15	0/10
Teicoplanin	0/16	0/15	0/10
Tetracycline	0/16	9/15	0/10
Linezolid	0/16	0/15	0/10
Gentamicin	1/16	7/15	1/10
Cotrimoxazole	0/16	5/15	0/10
Clindamycin	3/16	8/15	2/10
Cefazolin	3/16	13/15	3/10

## Data Availability

Data are available at the following link: URL https://osf.io/4nfxh/?view_only=33ef847b962a4282bd4273f502b15535 (accessed on 5 March 2024).
